# Rapid Determination of Xa Inhibitor Activity in Blood Using a Microfluidic Device that Measures Platelet Deposition and Fibrin Generation Under Flow

**DOI:** 10.1055/a-2547-5710

**Published:** 2025-03-25

**Authors:** Jason M. Rossi, Karen A. Panckeri, Soumita Ghosh, Tilo Grosser, Adam Cuker, Scott L. Diamond

**Affiliations:** 1 Department of Chemical and Biomolecular Engineering, Institute for Medicine and Engineering, University of Pennsylvania, Philadelphia, Pennsylvania, United States; 2 FloBio LLC, Philadelphia, Pennsylvania, United States; 3 Department of Medicine, Perelman School of Medicine, University of Pennsylvania, Philadelphia, Pennsylvania, United States; 4 Institute for Translational Medicine and Therapeutics University of Pennsylvania, Philadelphia, Pennsylvania, United States; 5 Department of Translational Pharmacology, Bielefeld University, Bielefeld, Germany; 6 Department of Pathology & Laboratory Medicine, Perelman School of Medicine, University of Pennsylvania, Philadelphia, Pennsylvania, United States

**Keywords:** oral anticoagulant, fibrin, thrombin, microfluidic

## Abstract

**Background:**

Patients taking direct oral anticoagulants (DOACs) often present complicated scenarios following major bleeding, stroke, or emergency surgery. Rapid whole blood assays of DOAC levels would aid clinical decisions such as the need for DOAC reversal.

**Methods:**

We developed a single-use, storage-stable, eight-channel microfluidic device to estimate factor Xa (FXa) inhibitor (apixaban or rivaroxaban) levels in venous thromboembolism or atrial fibrillation patients. The assay simultaneously measured whole blood clotting dynamics on collagen/tissue factor (TF; wall shear rate, 200 ^−1^ ) under four ex vivo conditions: no-treatment control, high dose Factor Xa inhibition, low dose or high dose FXa reversal agent (andexanet alfa). Fibrin and platelet deposition dynamics were monitored via two-color epifluorescence microscopy. Plasma samples were also evaluated by LC-MS/MS for DOAC concentrations.

**Results:**

Experiments with healthy volunteer blood spiked with DOAC verified device performance (DOAC IC _50_ ∼120 nM) and confirmed that andexanet alfa added to healthy donor blood had no off-target effect on platelet or fibrin signal. Patient whole blood monitored for 15 to 25 minutes (17 minutes mean runtime) allowed calculation of functional DOAC concentrations ranging from 2 to 500 nM that correlated well with LC-MS/MS determination of apixaban or rivaroxaban (R ^2^ = 0.7 or 0.9, respectively). Platelet dysfunction was not observed in any patient on DOAC. For a threshold of 100 nM DOAC, the area under the curve (AUC) was found to be 0.881 for apixaban and 0.933 for rivaroxaban.

**Conclusion:**

Microfluidic testing of whole blood can provide a rapid estimate of DOAC levels over the on-therapy range.

## Introduction

Since their regulatory approval, utilization of direct oral anticoagulants (DOACs) targeting either factor Xa (apixaban, rivaroxaban, edoxaban, betrixaban) or thrombin (dabigatran) has grown significantly for indications such as venous thromboembolism (VTE) and atrial fibrillation (AF) making them some of the most prescribed anticoagulants in use today. 1 2 3 When released to the market, their rapid onset, short half-life, reduced risk of major bleeding (particularly intracranial hemorrhage) and lack of need for routine laboratory monitoring were all significant advantages in comparison to vitamin K antagonists (VKAs). 1 4 5 6 Observational studies and clinical trials have revealed, however, that patients on DOACs can have a higher risk of major GI bleeding than those on warfarin, in certain cases. 7 8 9 Although their short half-life makes cessation of therapy a viable means to manage moderate bleeding risk, patients taking DOACs are still at increased risk of bleeding complications in emergency situations, unless steps are taken to actively reverse the anticoagulant effect. 10 11 Andexanet alfa is a modified, recombinant factor Xa molecule that binds and sequesters Xa-specific DOACs, preventing them from interacting with endogenous factor Xa. Although Andexanet alfa has been shown to be effective for reducing bleeding risk, prohibitive cost (up to $27,500 for low-dose therapy) and a lack of clinical evidence demonstrating superiority to four-factor prothrombin complex concentrate have limited its utilization. 12 13

Clinical scenarios where rapid measurement of DOAC is needed include: life-threatening bleeding, acute ischemic stroke, traumatic injury, and situations requiring urgent invasive procedures. 5 14 In these scenarios, rapid determination (<20 minutes) of DOAC presence, and estimated drug levels, would be clinically beneficial to determine the need for drug-specific reversal administration (Andexanet Alfa, Idarucizumab). In cases where the patient is unconscious and unable to identify the anticoagulant they are taking, a DOAC-specific test can inform the decision to use these specific reversal agents, rather than alternative therapies, such as fresh frozen plasma or four-factor prothrombin complex concentrate.

In these situations, conventional laboratory coagulation tests such as PT/INR and aPTT provide limited information and are not suitable for quantitative measurements. For example, at trough plasma concentrations, these tests may produce results that suggest no drug is present. 15 16 17 18 19 Thrombin time can be used for qualitative assessment of drug presence for direct thrombin inhibitors but is exquisitely sensitive even to very low, clinically inconsequential drug concentrations. 20 21 Similarly, heparin-calibrated anti-Xa tests can be used to screen for the presence of clinically relevant amounts of Xa inhibitors, but quantitation can vary significantly based on reagents and methods. 22 23 Other methods specific for measurement of certain DOACs, such as apixaban calibrated anti-Xa kits (for apixaban) and diluted thrombin time (for dabigtran), provide accurate drug concentrations, but have a slow turnaround, are not widely available in emergency settings, and are not designed for point of care (POC) use. 20 To date, only one automated hemostasis test has been authorized by the FDA for measurement of DOAC concentration, the HemosIL Liquid Anti-Xa test kit (Instrumentation Laboratories Co.), for apixaban. Another test, DOASense, has been CE-marked in Europe for the detection of DOACs using urine samples but provides no quantitative information.

Recent years have seen an influx of new potential POC devices for managing DOACs. Although many have shown success in detecting drug presence, none have demonstrated the ability to quantify drug concentrations. 24 25 26 27 28 Therefore, the need for a rapid, cost-effective method to detect and quantify DOACs in emergency situations remains. To potentially address this need, we deployed a whole blood microfluidic assay for measurement of oral factor Xa inhibitor that could be useful in a POC setting. In this study, we evaluated our rapid microfluidic assay against a reference standard method, liquid chromatography/tandem mass spectrometry (LC-MS/MS), in samples from DOAC-treated patients. This work represents an exploratory study to evaluate the capability of this microfluidic assay approach to detect, and potentially quantify Xa-inhibitors in whole blood. The system used in this work is composed of discrete OEM components and does not represent a product; this work was conducted as a means to evaluate and optimize the core assay technology. These components have a small footprint can be readily packaged into a single instrument and are amenable to deployment in a small bench space in a POC setting ( Supplementary Fig. S1 ).

## Material and Methods

### Device Design

**Fig. 1:**
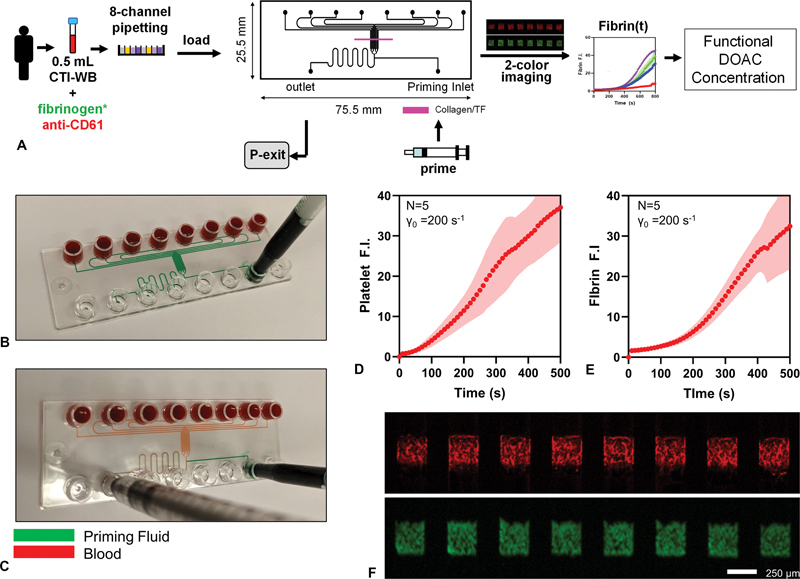
( **A** ) Schematic of microfluidic DOAC assay. Displacement of priming fluid ( **B** ) with blood upon starting the experiment ( **C** ). Average ( **D** ) platelet and ( **E** ) fibrin fluorescence intensity for *n* = 5 healthy donors, with *n* = 8 clots per donor, in chips prepared with collagen and tissue factor reaction stripes. ( **F** ) Pseudocolor fluorescence images for fibrin (red, AF594 fibrinogen) and platelets (green, AF488 mouse anti-human CD61).

A previously published eight-channel injection-molded device was redesigned such that all 8 sample wells were linearly arranged (“8 × 1”), facilitating multichannel pipetting to load the device ( Fig. 1A ). 29 Materials and assembly methodology were identical to the published device (see Supplementary Material ).

### Device Preparation and Flow Control

Microfluidic chips were assembled from injection-molded cyclic-olefin-copolymer (COC) components (ChipShop) as described previously. 29 Briefly, type-I fibrillar collagen (chrono-par, Chrono-Log) and lipidated TF (Dade Innovin, Siemens) were coated as a 250 µm wide stripe to an acrylic adhesive substrate (ThorLabs). Collagen was coated as provided by the manufacturer, and TF was diluted 1:50 in 0.1 µm sterile filtered water prior to coating. The adhesive was then laminated to the COC to complete the device. Blood was perfused through the chips by negative pressure applied at the outlet using a KPV14A diaphragm vacuum pump (Koge Electronics), maintaining constant pressure with a MFCS-EZ controller (Fluigent).

### Blood Collection

For healthy donor experiments, blood was collected from adults self-reporting as free from all medication for the preceding 10 days. Blood was collected via venipuncture into a 3 mL syringe containing corn trypsin inhibitor (CTI, Prolytix) at a final concentration of 40 μg/mL to minimize contact activation of FXII while allowing extrinsic pathway coagulation to proceed on the collagen/TF surface. For patient samples, blood was collected from adults (age ≥ 21 y), who were taking apixaban or rivaroxaban in steady-state for a history of VTE or AF ( Supplementary Fig. S2 ). Blood was drawn via venipuncture into “no additive” vacutainers (BD) and promptly transferred to a 15 mL centrifuge tube containing CTI (40 μg/mL). All blood was collected on-site at the Hospital of the University of Pennsylvania, and then either transported prior to analysis, with approximately 10–20 minutes between collection and experimentation (subjects 1–10), or tested on-site in the hospital (subjects 11–36).

The protocols for healthy donors and DOAC-treated patients were approved by the University of Pennsylvania Institutional Review Board. All subjects provided written informed consent. Upon consenting, demographic information, class of DOAC, dose, schedule, indication, and time of last DOAC administration were collected for each patient. ( Table 1 )

**Table 1:** Demographic and supplemental information for all subjects

Subject	M/F	Age	Drug	Indication	Dose schedule	Last dose (h)	Other med/comments
1	Male	68	Apixaban	VTE	2.5 mg BID	9	Ascorbic acid, glucosamine, **simvastatin** , cholecalciferol, fluticasone furoate/vilanterol, albuterol
2	Female	37	Apixaban	VTE	5 mg BID	3.5	**Tranexamic acid** , fumarate-FA, preanalytical clotting
3 a	Male	51	Apixaban	N/A	2.5 mg BID	4	**Pravastatin**
4 a	Female	44	Apixaban	VTE	5 mg BID	2.5	
5	Male	38	Rivaroxaban	VTE	20 mg OD	13.5	
6 a	Male	72	Apixaban	VTE	2.5 mg BID	12	Statin (unspecified)
7 a	Male	51	Apixaban	VTE	2.5 mg BID	2	Vitamin D
8 a	Female	50	Apixaban	VTE	2.5 mg BID	8	**Acetaminophen** , **aspirin** , buspirone, doxepin, duloxetine, gabapentin, hydrochlorothiazide, hydroxyzine, losartan, omeprazole, oxycodone, valacyclovir
9	Male	51	Apixaban	VTE	2.5 mg BID	15.5	Vitamin D, preanalytical clotting
10	Male	29	Rivaroxaban	VTE	10 mg OD	11	
11	N/A	N/A	N/A	N/A	N/A	N/A	Not provided, preanalytical clotting
12 a	Male	41	Rivaroxaban	VTE	2.0 mg OD	18	Vitamins
13 a	Female	60	Apixaban	VTE	5 mg BID	3	**Rosuvatatin** , **sertraline** , trazodone, vitamin D,melatonin
14	Female	69	Apixaban	AF	5 mg BID	14.5	**Atorvastatin** , budesonide/formoterol, albuterol, insulin, spironolacton, enalapril
15	Female	54	Rivaroxaban	VTE	10 mg OD	7	Aripiprazole, **fluoxetine** , bupropion
16	Female	26	Rivaroxaban		10 mg OD	21	Carvedilol, iron, gabapentin, hydroxychloroquine, mycophenolate, **sertraline**
17 a	Male	49	Rivaroxaban	VTE	20 mg OD	21.5	
18 a	Female	31	Apixaban		2.5 mg BD	7.5	**Fluoxetine** , levonorgestrel, vitamins, formoterol fumerate, lisdexampfetamine
19 a	Female	29	Rivaroxaban	VTE	10 mg OD	22.5	**Acetaminophen** , **sertraline** , tramadol, mirena
20	Female	30	Apixaban	VTE	2.5 mg BID	3	**Tranexamic acid**
21	Female	79	Apixaban	VTE	5 mg BID	9	Albuterol, **atorvastatin** , tafluprost, hydrochlorothiazide, levothyroxine, loratadine, metoprolol, quinapril
22 a	Female	50	Apixaban	VTE	5 mg BID	1.5	**Aspirin** , **acetaminophen** , lisinopril, cetrizine
23 a	Female	35	Rivaroxaban	VTE	20 mg OD	18.5	Montelukast, norethindrone, tacrolimus, fluticasone, vilanterol
24 a	Female	28	Apixaban		2.5 mg BID	7.5	Clindamycin, phenazopyridine, spironolactone, triamcinolone
25 a	Male	57	Rivaroxaban		10 mg OD	5.5	Semaglutide, isinopril, albuterol, insulin, **atorvastatin** , metformin, **ticagrelor**
26 a	Female	61	Rivaroxaban	VTE	10 mg OD	17	Cetirizine, clonazepam, rizatriptan, **sertraline** , **simvastatin**
27 a	Female	22	Rivaroxaban	VTE	20 mg OD	21.5	Progestin
28	Female	63	Apixaban	VTE	5 mg BID	9	Estradiol, levalbuterol, hydroxyurea, magnesium chloride, losartan, montelukast, dexlansoprazole, praluent, fremanezumab, folic acid, nebivolol
29 a	Female	34	Apixaban	VTE	2.5 mg BID	25	
30 a	Male	29	Apixaban	VTE	2.5 mg BID	N/A	Aripiprazole
31 a	Female	26	Apixaban	VTE	5 mg BID	4.5	Etonogestrel
32	Female	72	Rivaroxaban	VTE	10 mg OD	4	
33	Female	37	Rivaroxaban	VTE	10 mg OD	20.5	Ferrous sulfate, **transexamic acid**
34	Female	66	Apixaban	VTE	2.5 mg BID	4.5	**Atorvastatin**
35	Female	22	Rivaroxaban	VTE	10 mg OD	24.5	Duloxetine, levonorgestrel, spironolactone
36 a	Female	59	Apixaban	VTE	5 mg BID	4	Esomeprazole, gabapentin, hydroxychloroquine, insulin, metropolol, modafinil, mirabegron, solifenacin, eletriptan

Abbreviations: AF, atrial fibrillation; VTE, venous thromboembolism.

Entries of “N/A” indicate the information was omitted or never collected from the subject. Medications known to modulate hemostasis are bolded. OD and BID indicate medications taken once daily and twice daily. Final counts were *n* = 11 males (average age = 48.7) and *n* = 24 females (average age = 45.2), with information not collected for *n* = 1 subject.

a Patients had both microfluidics runs without error and LC-MS/MS data available.

### Platelet and Fibrin Detection During Clotting

Platelets were labeled in whole blood by the addition of an AlexaFluor 488-conjugated anti-CD61 molecule (Bio-Rad Laboratories) and fibrin was quantified via the addition of AlexaFluor 594-conjugated fibrinogen (Life Technologies Co.), as previously described. 29 Chips were mounted on a Lumascope 620 (Etaluma Inc.) LED epifluorescence microscope with a 2.5× objective (Meiji Techno). Each chip was primed with 1% bovine serum albumin (BSA) in HEPES-buffered saline (HBS) to passivate the surfaces ( Fig. 1A ). A volume of 60 μL of whole blood was added to each inlet well. A vacuum pressure of 140 mbar was applied to the outlet to yield an initial wall shear rate of 200 s ^−1^ . Fluorescence images were captured at 10 s intervals for green and red LED channels to obtain kinetic data for platelet and fibrin deposition on the collagen/TF zone for up to 25 min. Images were analyzed with ImageJ to calculate the average pixel brightness of an inner 200 μm by 200 μm region within each clot.

**Fig. 2:**
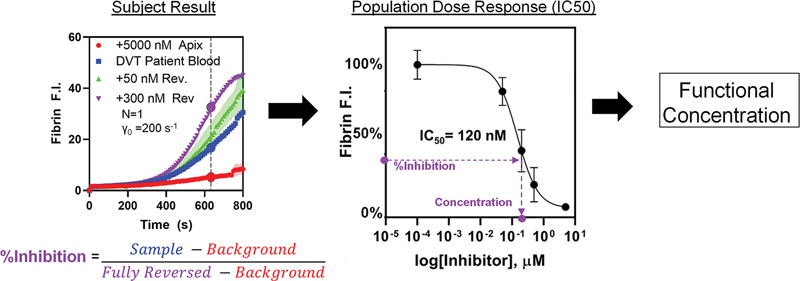
Outline of the process for translating relative fibrin fluorescence intensity signals to an approximate concentration of DOAC, using an IC50 curve generated from healthy donors free from known diseases or medications. A unique IC50 curve was generated for apixaban and rivaroxaban in previously published results.

### Assessment of Reversal Agent Behavior in the Absence of DOAC

Tests were run with blood from healthy donors (no DOAC) to verify that the only effect of adding the reversal agent, andexanet alfa (AstraZeneca), ex vivo was to target DOAC activity rather than impacting clotting in a DOAC-independent manner. Whole blood from healthy donors was tested with andexanet alfa, added from a 0.25 mg/mL stock in HBS, which was prepared from a 10 mg/mL stock in the manufacturer's buffer. For *n* = 3 healthy donors, whole blood was combined with the andexanet alfa to a final concentration of 50 or 300 nM. HBS was added such that whole blood comprised 90% of the final volume. The andexanet alfa-treated blood was added to the microfluidic device and the assay was performed as described previously.

### DOAC Reversal and Detection

For DOAC-treated patients on either rivaroxaban or apixaban, blood samples were obtained and analyzed, using the microfluidic device to measure platelets and fibrin deposition under flow as described above. To reverse the fibrin inhibition in patient blood containing a factor Xa inhibitor, andexanet alfa was added to four of the eight wells of the device: two wells with a final concentration of 300 nM, and two with a final concentration of 50 nM. The 300 nM dose was expected to fully reverse most doses of Xa inhibitors that patients are likely to be on, and the 50 nM dose was included to potentially provide more precise results for patients whose plasma DOAC levels were very low. To provide a “fully inhibited” fibrin response, a supratherapeutic dose of Xa inhibitor, either rivaroxaban or apixaban to match the patient's prescription, was added to two of the channels. Rivaroxaban or apixaban (SelleckChem) was dissolved in dimethyl sulfoxide (DMSO, 10 mM stock solution) and diluted to 100 μM in HBS, then added to a final concentration of 5,000 nM. Finally, the remaining two wells were run as collected from the patient, without further modification (“no-treatment control”). For all wells, “make-up” HBS volume was added such that blood comprised 90% of the final volume of the mixture to ensure no bias in the hematocrit across the drug conditions. For subjects 1 through 19, fluorescence data were collected for 15 minutes. For all subsequent patients, data were collected for 25 minutes, or until channel occlusion was observed.

### Calculation of Effective DOAC Concentration

To calculate the concentration of DOAC in each patient's blood, the relative fluorescence intensity of the patient's unmodified blood and the high (+300 nM andexanet alfa) reversal agent condition were compared. These were then scaled to a previously measured dose response of the fibrin fluorescence intensity for apixaban and rivaroxaban added in vitro to medication-free donor whole blood. 29 For the purposes of this scaling, any fluorescence in the 5,000 nM anticoagulant condition was considered a background signal. According to a three-parameter sigmoid fit of the IC50 curve ( Fig. 2 ; Supplementary Fig. S3 ), the concentration of DOAC in the patient sample was interpolated using the healthy donor IC50 data, by identifying the concentration of drug that demonstrated an equivalent percent reduction in fibrin fluorescence relative to a fully reversed/no drug condition. A detailed description of this method is available in Supplementary Materials .

### Data Screening

Since this study was the first deployment of this technology in a clinical setting, efforts were made to optimize assay performance and data quality, particularly issues of preanalytical variability that were encountered. Subjects were excluded from further analysis if any of the following data was missing: completed microfluidic assay results (all wells of the device, and comparison between duplicate wells), full clinical labs (CBC, blood chemistry; see Supplementary Table S2 ), and LC-MS/MS results. Blood samples that clotted during transport ( *n* = 3) could not be run on the device and therefore did not have microfluidic assay results. Runs for *n* = 13 apixaban patients and *n* = 7 for rivaroxaban patients passed all acceptance criteria and were used for all subsequent analyses ( Supplementary Fig. S4 ).

### LC-MS/MS Drug Level Detection

For each subject tested with the microfluidic device, the remaining whole blood was combined with sodium citrate (0.11 M) at a ratio of 9:1. Samples were spun down into platelet-poor plasma via centrifugation at 2,000 g for 20 min, and stored at −80°C. In three cases (Subjects 2, 9, and 11), preanalytical clotting prevented a plasma sample from being obtained, and in one other case (Subject 1), no plasma sample was collected. Plasma samples were analyzed for DOAC concentration in two batches: initial subjects who were on apixaban ( *n* = 5) were analyzed as LC-MS/MS batch 1, and subjects on both apixaban and rivaroxaban ( *n* = 15) were analyzed as batch 2, separately. Subjects from batch 1 on rivaroxaban were not tested due to the specificity of the LC/MS-MS method for apixaban. LC-MS/MS procedures are described in Supplementary Materials .

### Statistical Analysis

Statistical significance was calculated for paired *t* -tests, using GraphPad Prism 9 (GraphPad Software). Shaded error regions on plots represent the standard deviation from the mean. For the evaluation of the coefficient of variation (CV) for healthy donors, fluorescence intensity at 400 s was evaluated. For DOAC patient data, fluorescence intensities are reported for a single timepoint, at the time when the difference between the average fluorescence intensity of the unmodified patient blood and the average of the 300 nM andexanet alfa treated blood was found to be maximal. Where indicated, data for each patient were normalized to the average fluorescence intensity of the 300 nM andexanet alfa response. Ninety-five percent confidence bands of the best-fit line were reported for the linear regression of LC-MS/MS concentration versus microfluidic functional concentration using GraphPad Prism. Fits were not required to go through the origin.

## Results

### Healthy Whole Blood Clotting on Collagen/TF

To confirm the performance of the “8 × 1” chip, whole blood from *n* = 5 healthy donors was perfused through all 8 wells of the devices prepared as described, at 200 s ^−1^ ( Fig. 1D, E ). Across the five healthy donors, the platelet signal showed an average interdonor CV of 25%, with the fibrin signal having a CV of 20% at 500 s. No correlation was observed between the platelet or fibrin signals and the specific well, 1 2 3 4 5 6 7 8 suggesting flow in each channel was identical.

**Fig. 3:**
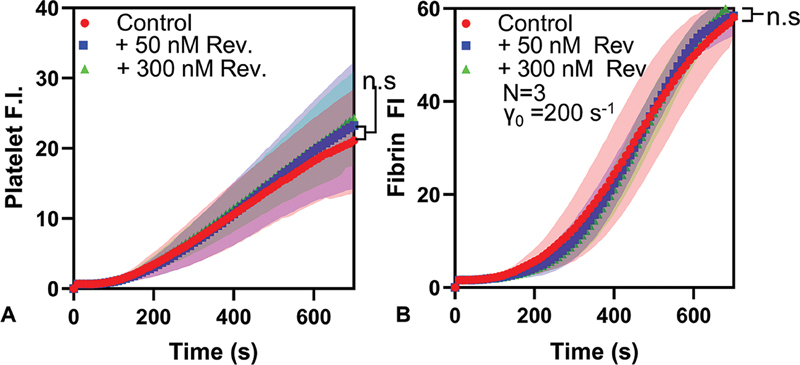
Fluorescence signal for ( **A** ) platelets and ( **B** ) fibrin for healthy donor blood with andexanet alfa added. No anticoagulant-independent effect on either signal was observed across 3 healthy donors, implying that when the reversal agent is used with blood from DOAC-treated patients, any increase in the signal can be attributed to the functional reversal of the drug action.

To verify that andexanet alfa alone does not interfere with normal clotting behavior, healthy donor blood was combined with a reversal agent in the absence of an Xa inhibitor. For *n* = 3 donors, no significant difference ( *p* > 0.05) in average signal was observed between blood with and without reversal agents in either the platelet or fibrin signal ( Fig. 3 ).

### DOAC Patient Data

**Fig. 4:**
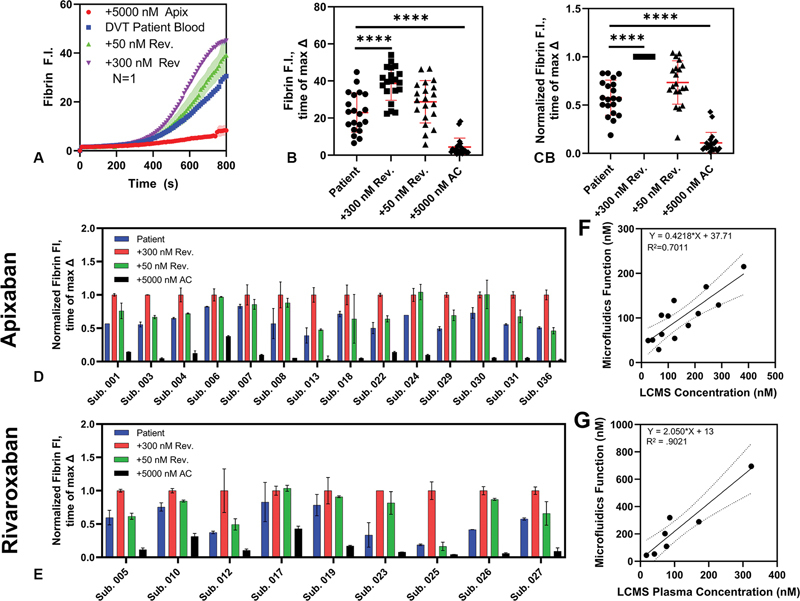
( **A** ) Example subject fibrin fluorescence signal for the microfluidic assay. Raw ( **B** ) and normalized ( **C** ) fibrin fluorescence intensities for 20 included subjects. ( **D, E** ) FI for all conditions normalized to the fully reversed lanes for all subjects. ( **F, G** ) Correlation between measured functional concentration and plasma concentrations as detected by LC-MS/MS (subjects 001, 005, and 010 did not have LC-MS/MS data measured and are not represented).

To assess the ability of our assay to rapidly detect DOAC presence in patients on apixaban or rivaroxaban, the test utilized small volumes of andexanet alfa to restore fibrin activity which could then be compared to each patient's inhibitor-containing blood sample. For *n* = 13 apixaban and *n* = 7 rivaroxaban patients with complete microfluidic assay data, clinical labs data, and LC-MS/MS data, consistent, significant restoration of the fibrin signal was observed ( *p* < 0.0001) for the addition of 300 nM of andexanet alfa, relative to their unmodified sample ( Fig. 4B, C ). It was assumed that 300 nM was sufficient to provide either an approximately equimolar ratio or an excess of reversal agent relative to inhibitor concentrations expected for most patients on DOACs. 30 31 32 In most cases, the 50 nM concentration of the reversal agent provided only a partial restoration of fibrin fluorescence ( Fig. 4B ). For patients with low levels of DOAC detected in their plasma samples, no difference was observed between the 300 and 50 nM reversal conditions ( Fig. 4D, E ). This indicates that the low reversal condition has limited utility and would not be required for future studies, and confirms that 300 nM and andexanet alfa have no confounding effects when with represents a significant molar excess over the Xa inhibitor present. All subjects demonstrated nearly fully suppressed fibrin signal for the 5,000 nM matching apixaban or rivaroxaban DOAC sample, with any remaining signal likely due to fluorescent fibrinogen binding to platelets ( Fig. 4B, C ).

Previous studies using in vitro spiked whole blood from healthy donors showed maximal differentiation between DOAC-inhibited and unmodified fibrin channels at around 500 s ( Supplementary Fig. S5 ). 29 With the greater level of variance in both the patient characteristics and in the timing between blood collection and assay execution for these patients, however, the timescale of clotting was found to vary more than in healthy donors. Therefore, fibrin fluorescence intensities were evaluated for each patient at the timepoint where the difference between the high (+300 nM) reversal agent condition and the subject's unmodified blood sample was at a maximum (referred to as “time of max Δ”), rather than at 500 s. The average time of max Δ observed across all patients was 17 min. No single, patient-linked variable investigated was found to be predictive of the time of the max Δ ( Supplementary Fig. S6 , Supplementary Table S2 ), but the various concomitant medications each patient was taking may have contributed to fibrin signal variation.

For the 20 patients with data in the study, the raw fibrin fluorescence intensity (FI) evaluated at the time of max Δ showed a statistically significant difference ( *p* < 0.0001) between the patient's blood with fluorescent labels only and the high reversal condition ( Fig. 4B ). Although the relative values of these two conditions were similar between the subjects, a wide range of fluorescence magnitudes were observed. To facilitate visualization of the effect of the reversal agent, data for all conditions was also normalized to their fully reversed condition. For each patient, the average fibrin signal for the two high reversal (+300 nM andexanet alfa) lanes was used to scale all data for that patient. With this modification, it is readily apparent that all subjects showed restoration of fibrin signal with the addition of reversal agent ( Fig. 4C ). Since, for the purpose of the DOAC calculation, each patient's fibrin data is evaluated against their reversed blood rather than a global value, this normalized data shows the range of behavior more effectively. Overall, despite expected patient variations in the initiation time and final extent of fibrin production, spiking with high-dose DOAC always quenched fibrin generation, while spiking with high-dose reversal agents never decreased the amount of fibrin made.

Contrary to the fibrin signal, platelet FI was largely independent of the presence of a reversal agent. A small, but statistically significant, response was found between the patient's Xa inhibitor-treated blood and the high reversal condition ( *p* = 0.001, Supplementary Fig. S7B ). Comparatively, a larger difference ( *p* < 0.0001) was observed in comparing the patient's Xa inhibitor-treated blood (“patient”) and fully inhibited condition (“ + 5,000 nM Rev”), indicating platelets are most impacted when thrombin is abolished completely ( Supplementary Fig. S7B ).

### Functional DOAC Concentration

Across the patients, a broad range of drug concentration values within the therapeutic window were calculated. Predictions ranged from 20 to 500 nM ( Fig. 4F, G ). Predicted concentrations correlated well with the time since the last dose reported by the patient (R ^2^ = 0.93) for subjects on rivaroxaban, but very poorly (R ^2^ = 0.003) for subjects on apixaban ( Supplementary Fig. S8 ).

### Plasma Concentrations Quantified by LC-MS/MS

LC-MS/MS data was obtained using citrated plasma samples, spun down from the same whole blood samples used for the chip assay. Linear regression was performed to compare the functional concentration from the microfluidics assay and the concentration reported from LC-MS/MS. For apixaban, an R ^2^ of 0.70 was found for the correlation ( Fig. 4F ). For rivaroxaban, an R ^2^ of 0.90 was found, indicating a reasonable correlation between LCMS and the assay for drug concentration, for this relatively small dataset used to demonstrate the feasibility of the microfluidic approach ( Fig. 4G ).

ROC analysis for the functional concentration predictions correctly classifying DOAC concentration as above or below thresholds was performed, in reference to the LC-MS/MS measurements. For a threshold of 100 nM, the area under the curve (AUC) was found to be 0.881 for apixaban, and 0.933 for rivaroxaban ( Supplementary Fig. S9 ). For a threshold of 60 nM, the AUCs were found to be 0.91 and 1, respectively ( Supplementary Fig. S9B, D ).

## Discussion

Although DOACs are generally considered to have a superior safety profile to older classes of anticoagulants, the lack of development of a rapid diagnostic for the detection and quantification of DOACs in acute clinical scenarios is now recognized as an unmet need. 3 33 34 35 The need for rapid testing is particularly relevant in situations such as cases of major bleeding, in advance of thrombolytic treatment for ischemic stroke, in cases of traumatic injury, and in cases of urgent invasive surgery. 3 36 37 A rapid bedside DOAC test would be beneficial from a medical facility cost-benefit perspective since DOAC reversal agents can cost as much as $25,000 per dose. In addition, improved testing may reduce the risk of patient complications from bleeding, which can substantially increase the overall cost of care, and reduce hospital performance metrics.

In acute testing scenarios where triage decisions often are being made in <20 minutes, test methods that are precise but slow (or not widely available) are impracticable. Liquid anti-Xa assays have been shown to correlate very well with LC-MS/MS measurements of plasma levels of factor Xa inhibitors, but these assays are relatively expensive and are not designed for POC use, even when available in hospital laboratories. 37 38 39 For these reasons and others, only approximately 1% of labs that perform hemostasis testing in the United States perform quantitative DOAC testing. 40

The assay described in this work would allow for rapid assessment of both platelet and fibrin function in approximately 15 minutes under whole blood flow at venous shear rates, using small volumes of blood (<400 μL). Anticoagulation in patients on DOAC was reversed on-chip, with small amounts (∼1 μg) of reversal agent. Restoration of the fibrin signal with the addition of a reversal agent was observed for all ( *n* = 20) subjects. This, combined with the lack of nonspecific response induced by andexanet alfa ( Fig. 3 ), suggests that DOAC was detectable in all patients tested. Consistent detection of DOAC presence represents an improvement on common methods such as PT/aPTT for Xa inhibitors (where sensitivity/specificity can be in the range of 50% depending on the thromboplastin used), and on previously published POC devices. 24 25 27 41 Threshold analysis around relevant concentrations of each drug for perioperative management, produced ROCs > 0.88 for both drugs at both 60 and 100 nM, suggesting this assay may be able to provide rapid insight relative to clinical decision points, particularly compared with other testing strategies. 42 The number of samples included in each ROC analysis was very small, however, and so further study is required.

Although full quantitative agreement with the LC-MS/MS is desirable, in acute cases, a POC test that provides semi-quantitative information on whether a subject is above or below the ISTH threshold levels still provides significant clinical benefit. Guidance from the Anticoagulation Forum for DOAC-treated patients with major bleeding suggests that administration of a reversal agent should only proceed if clinically relevant concentrations of DOAC can be measured, or are strongly suspected. 43 Similarly, prior to invasive procedures, demonstration (or reasonable expectation) of plasma DOAC levels that are above the threshold is considered a primary factor in the decision to deliver a reversal agent or not.

The processing of fluorescent image data and the mathematical interpretation of the results of the present assay are not complex and can be computed in a matter of seconds. Since the data collected from the microfluidic device represents a complete picture of physiological hemostasis (including fluid flow, collagen, TF, platelets, and fibrin), we suggest that further development of this assay, microfluidic device, and analytical techniques will result in the potential for a rapid (<15 minutes) POC diagnostic device for determining DOAC presence and levels.

Finally, the reasonable correlation (R ^2^ = 0.7) with plasma concentrations reported by LC/MS-MS for apixaban, and (R ^2^ = 0.9) for rivaroxaban suggests that a more refined device, analytical methods, or alternative data output metrics could ultimately provide semi-quantitative DOAC drug levels. The limited orthogonality observed between LC-MS/MS may be due in part to the fact that the reference data for the IC50 curves considered concentrations in whole blood, while the LC-MS/MS data reports concentrations in plasma after a solid phase extraction. The high plasma protein binding for both apixaban and rivaroxaban could possibly lead to systematic differences between DOAC plasma concentration, and the effective concentration displaceable by reversal agent. 44 45 Although there is a clear correlation, more study is required to elucidate the proportionality between microfluidics function concentrations and measure drug concentrations.

Before deployment as an in vitro diagnostic product in clinical settings, there are several key aspects of the microfluidic assay in its current form that will have to be addressed. First, sample collection would need to be modified to fit into hospital workflow. Anti-coagulant compatibility is a limitation of the assay. Blood is currently collected into CTI to allow extrinsic coagulation to proceed without the sample activating during handling. This anticoagulant is not widely available in vacutainers, however, and has a relatively short working time before nonspecific sample activation. More common anticoagulants with significantly longer working times, such as sodium citrate and lithium heparin, are widely available in hospitals, but require additional reagents to restore coagulation for testing: calcium ions for citrate, and protamine sulfate for heparin. In both cases, the performance of the blood after treatment is not necessarily representative of an unmodified blood sample. Additionally, andexanet alfa can potentially indirectly impact the activity of heparin through an interaction with antithrombin. It is possible that citrated samples can be used for this assay, but further research is required to evaluate the impact on the assay. An antidote specific to the direct thrombin inhibitor, dabigatran, has been approved for use (idarucizumab). Using this material, the testing strategy described may be extended to dabigatran for future work. By combining both reversal agents on a single device, simultaneous classification and quantification of DOACs may be possible in a POC setting.

## Conclusion

The assay presented in this work shows the potential to provide actionable information on the concentration of Xa inhibitors present in a patient's whole blood sample, in a point-of-care setting in under 20 minutes. A larger study with more patients is required to determine the quantitative precision, but this small study has successfully demonstrated feasibility. Further refinement to the assay will be required to make it practicable in a hospital POC setting—either by incorporating more liquid-handling functionality onto the chip itself to enable the use of no-additive vacutainers, or by adapting the assay to work with sodium citrate.
