# Supplementary material for: Rapid Determination of Xa Inhibitor Activity in Blood Using a Microfluidic Device that Measures Platelet Deposition and Fibrin Generation Under Flow
Source: TH Open. 2025 Mar 25;9:a25475710. doi: 10.1055/a-2547-5710 (PMC11967380; doi:10.1055/a-2547-5710)
Supplement: Supplementary file 1 — Supplementary Material [file 10-1055-a-2547-5710-s25010002.pdf]

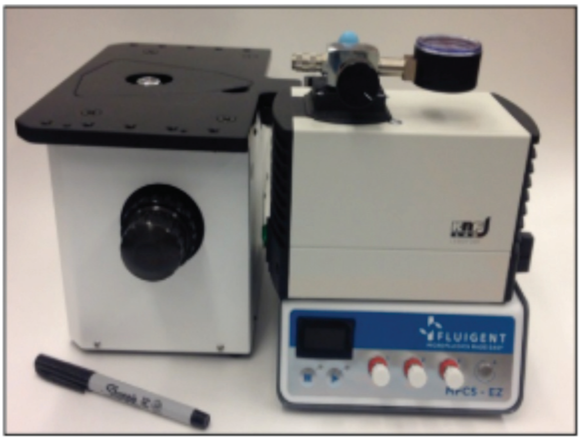

Supplementary Fig. S1 Footprint of LED microscope, flow controller, and vacuum pump. Benchtop space requirement is comparable to point-of-care instruments.

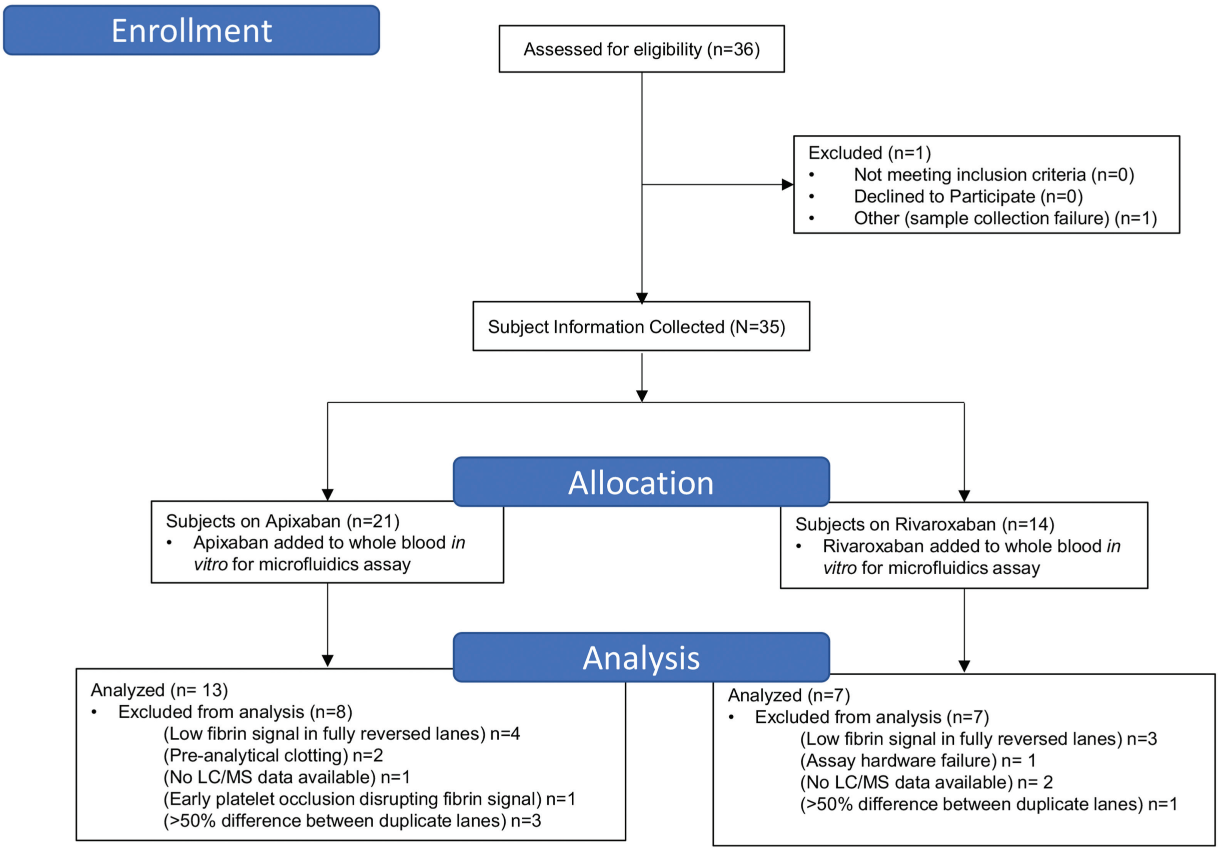

Supplementary Fig. S2 Consort-style diagram for patient flow data.

 © 2025. The Author(s).

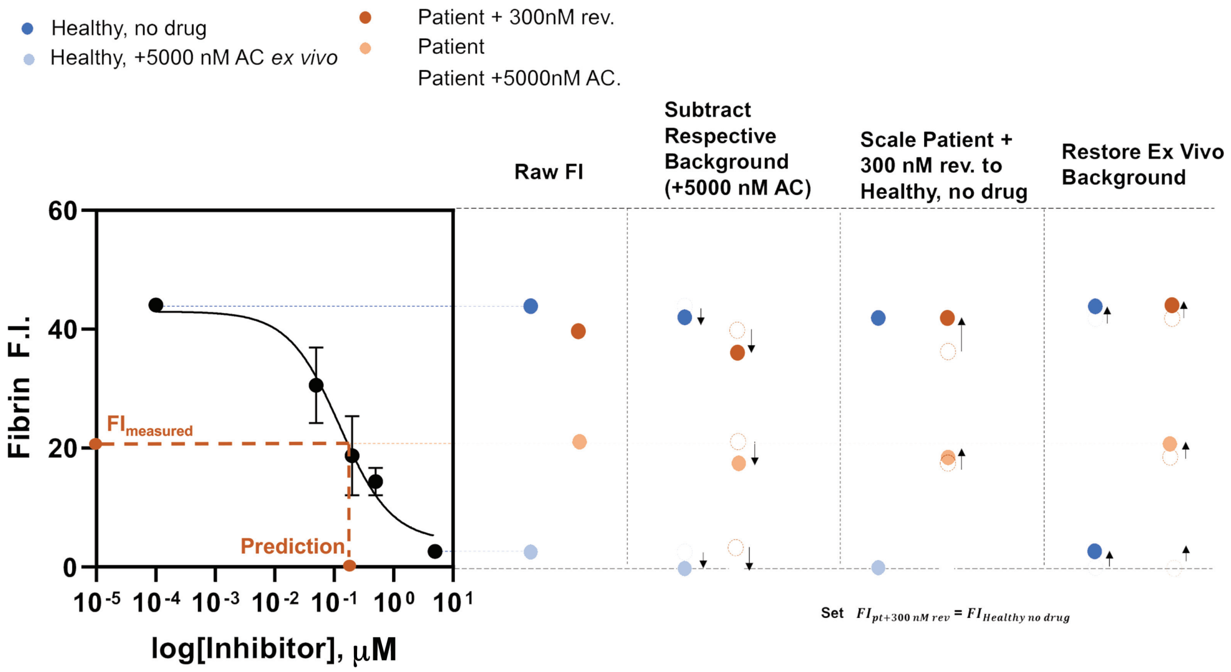

Supplementary Fig. S3 Procedure for generating a prediction for the functional concentration of DOAC using microfluidic data and IC50 data from healthy individuals.

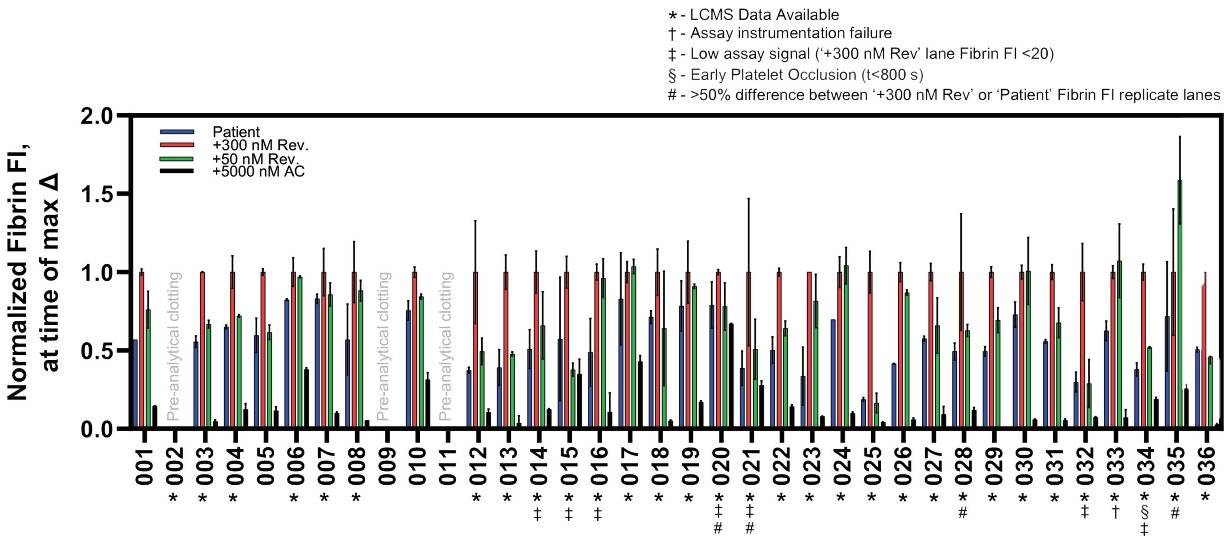

Supplementary Fig. S4 Summarized fibrin results for all patients with annotations for cases where the data was not included in the comparison with LC/MS.

 © 2025. The Author(s).

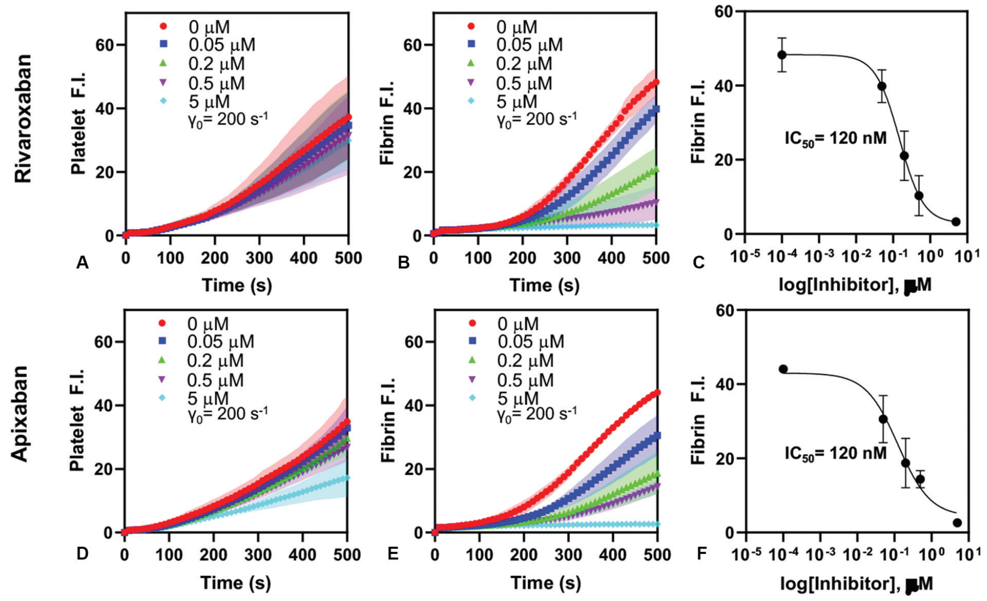

Supplementary Fig. S5 Microfluidics fluorescence and IC50 data for healthy adult blood spiked with rivaroxaban (**A–C**) or apixaban (**D–F**), previously published (Reprinted with permission from AIP Publishing, Jason M. Rossi, Scott L. Diamond; Scalable manufacture of a disposable, storage-stable eight-channel microfluidic device for rapid testing of platelet, coagulation, and drug function under whole blood flow. Biomicrofluidics 2020; 14(05):054103).

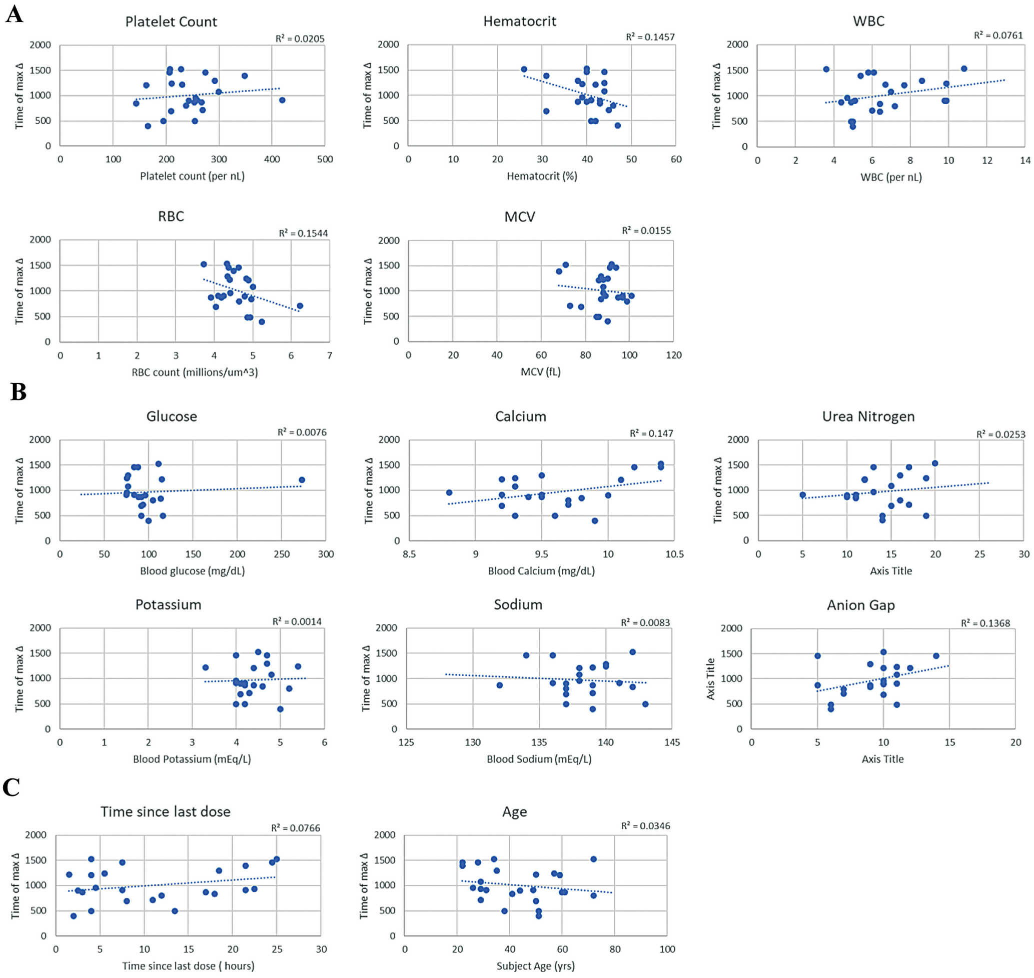

Supplementary Fig. S6 Blood cell count (**A**) and chemistry (**B**) results, and (**C**) other metrics for 20 included patient datasets, plotted against the time where the fibrin signal for the high reversal agent and unmodified blood lanes were maximally differentiated. No significant correlations were found for these variables.

 © 2025. The Author(s).

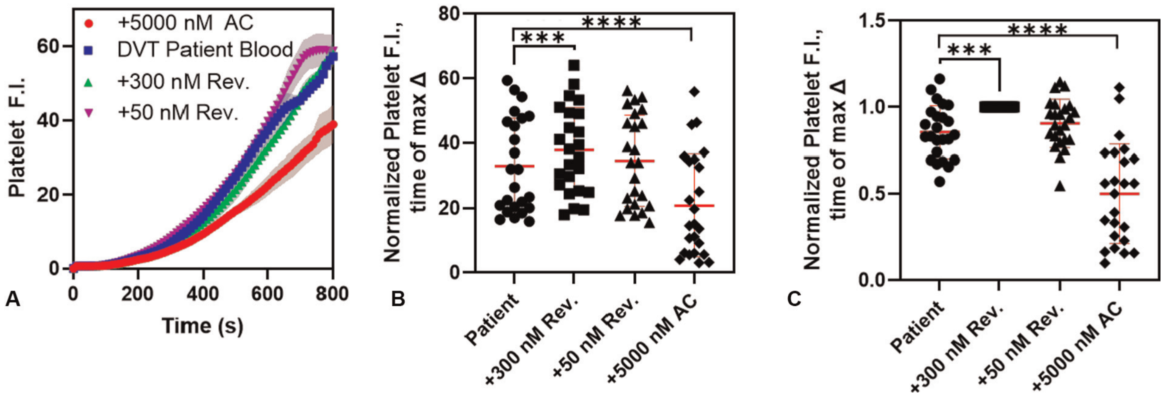

Supplementary Fig. S7 Summarized Platelet data for all patients included LC/MS and microfluidics functional concentration comparison, including an individual result (**A**), raw fluorescence (**B**), and normalized fluorescence (**C**). $^{***}p < 0.001$.

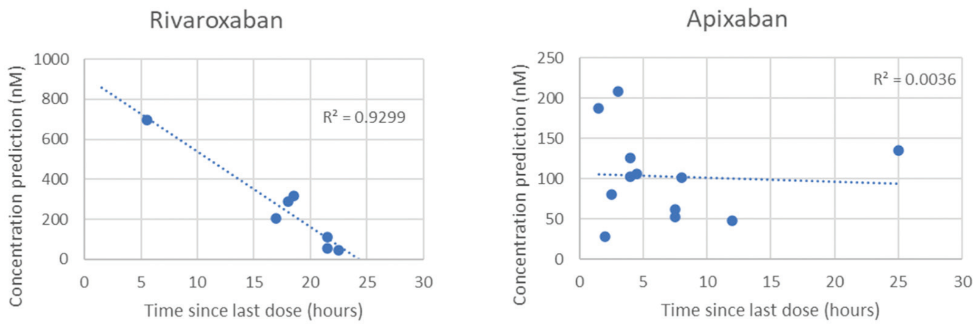

Supplementary Fig. S8 Correlation between LC-MS/MS concentration and time since last dose.

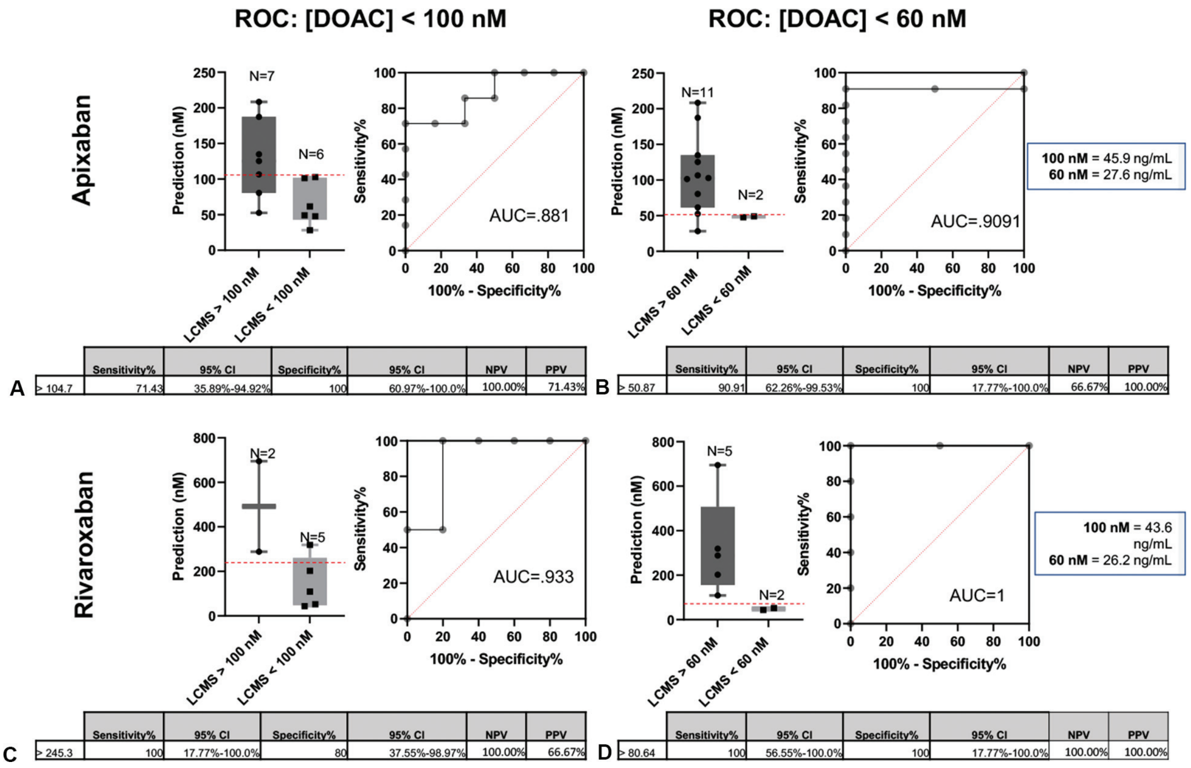

	Sensitivity%	95% CI	Specificity%	95% CI	NPV	PPV
A > 104.7	71.43	35.89%-94.92%	100	60.97%-100.0%	100.00%	71.43%
B > 50.87	90.91	62.26%-99.53%	100	17.77%-100.0%	66.67%	100.00%
C > 245.3	100	17.77%-100.0%	80	37.55%-98.97%	100.00%	66.67%
D > 80.64	100	56.55%-100.0%	100	17.77%-100.0%	100.00%	100.00%

Supplementary Fig. S9 ROC analysis for correction determination of Xa inhibitor concentrations as being above or below threshold values of 100 or 60 nM. Sensitivity and specificity reported in ►**Supplementary Fig. 10** are reported at the cut-off functional concentration value which provided the largest sum of sensitivity and specificity together. Positive predictive value (PPV) and negative predictive value (NPV) are reported for the same cutoff values.

 © 2025. The Author(s).

Nominal Calibration Level(µg/mL)	Measured Conc. (µg/mL)	Accuracy (%)
**0.0003**	0.000321	107
**0.001**	0.00108	108
**0.003**	0.00307	102
**0.01**	0.00950	95.0
**0.03**	0.0258	85.9
**0.1**	0.0993	99.3
**0.3**	0.308	103
**1.0**	0.996	99.6

QC Level	Low	Mid	High
**Nominal (µg/mL)**	**0.003**	**0.03**	**0.3**
**Measured Conc. (µg/mL)**	0.00354	0.0301	0.341
	0.00303	0.0298	0.332
	0.00343	0.0303	0.337
	0.00328	0.0306	0.346
	0.00316	0.0311	0.325
	0.00301	0.0303	0.326
**Average (µg/mL)**	0.00324	0.0304	0.335
**Accuracy (%)**	108	101	112
**CV (%)**	6.6	1.5	2.5

A

Nominal Calibration Level(µg/mL)	Measured Conc. (µg/mL)	Accuracy (%)
**0.0003**	0.000355	118
**0.001**	0.000926	92.6
**0.003**	0.00321	107
**0.01**	0.00921	92.1
**0.03**	0.0278	92.7
**0.1**	0.0934	93.4
**0.3**	0.313	104
**1.0**	0.996	99.6

QC Level	Low	Mid	High
**Nominal (µg/mL)**	**0.003**	**0.03**	**0.3**
**Measured Conc. (µg/mL)**	0.00246	0.0319	0.308
	0.00268	0.0315	0.332
	0.00305	0.0306	0.318
	0.00267	0.0313	0.312
	0.00322	0.0323	0.315
	0.00254	0.0318	0.326
**Average (µg/mL)**	0.00277	0.0316	0.319
**Accuracy (%)**	92.3	105	106
**CV (%)**	10.8	1.9	2.8

B

Supplementary Fig. S10 Method qualification results for LC-MS/MS procedure for the second plasma sample batch, for (**A**) rivaroxaban and (**B**) apixaban.

Supplementary Table S1 Settings for Mass Spec procedure used for the second batch of plasma samples from DOAC patients

Test Article	+/–	Q1	Q3	DP	EP		CE	CXP	IS
Apixaban	+	460.2	199.1	106	10		50	5	5,500
Apixban-$^{13}CD_3$	+	464.3	203.3	110	10		51	5	5,500
Rivaroxaban	+	436.1	145.2	98	10		35	9	5,500
Rivaroxaban-d_4	+	440.2	145.2	93	10		35	4	5,500

 © 2025. The Author(s).

Supplementary Table S2 Standard blood chemistry and cell count results for all subjects. Red values were flagged as high by the laboratory, blue values were flagged as low

Subject	Glu	Urea nit	Creat	Na	K	Cl	CO2	An gap	Ca	WBC	RBC	Hemoglobin	Hematocrit	RDW	MCH	MCHC	MCV	Plat	DOAC
1	66	26	1.18	142	4.4	107	26	9	8.9	5	4.95	15.1	44	13.3	31	34	89	176	A
2																			A
3	92	19	1.12	137	4	101	30	6	9.6	5	4.87	14.3	41	12.3	29	34	85	195	A
4	96	10	0.88	137	4.1	104	23	10	10	9.9	4.79	14.6	43	13.7	31	34	89	243	A
5	116	14	0.92	143	4.2	106	26	11	9.3	4.9	4.94	14.7	42	12.8	30	35	86	254	R
6	105	16	0.89	137	5.2	103	27	7	9.7	7.2	4.65	15.7	46	13.2	34	34	99	238	A
7	100	14	0.92	139	5	103	30	6	9.9	5	5.24	16.1	47	12.6	31	34	90	166	A
8	92	15	1.18	137	4.1	100	27	10	9.2	6.4	4.04	10	31	19	25	32	78	209	A
9	84	18	1.01	139	4.9	102	31	6	9.7	7.8	5.59	16.8	49	12.6	30	34	88	385	A
10	94	17	1.2	139	4.3	104	28	7	9.7	6	6.23	14.6	45	15	24	32	73	269	R
11																			
12	114	11	0.96	142	4.6	104	29	9	9.8	6.4	4.96	15.2	43	13.2	31	35	87	144	R
13	89	11	0.72	139	4.4	103	27	9	9.5	4.9	3.92	13	38	13.4	33	34	97	253	A
14										13	5.11	15.3	46	17.2	30	33	91	273	A
15	79	14	0.98	140	4.3	102	28	10	9.5	5	4.85	15.1	42	13.4	31	35	87	248	R
16										6.4	4.51	11.7	38	15.2	25.9	30.8	84	286	R
17	84	11	0.96	141	4.2	102	29	10	9.2	5.1	4.25	14.4	41	13.7	34	35	97	259	R
18	75	5	0.61	136	4	102	23	11	9.5	9.8	4.1	14.6	41	13.2	36	35	101	419	A
19																			R
20	24	8	0.68	142	4.6	102	25	15	9.6	5.2	4.25	12.8	38	13.1	30	33	90	281	A
21																			A
22	115	12	0.88	139	3.3	101	26	12	9.2	6.7	4.4	13.2	39	15.5	30	34	88	230	A
23	77	16	1.12	140	4.7	106	25	9	9.5	8.6	4.35	12.7	38	13	29	33	87	292	R
24	84	17	0.87	134	4	96	24	14	10.2	5.8	4.37	13.7	40	12.7	31	34	91	274	A
25	76	19	0.97	140	5.4	102	27	11	9.3	9.9	4.84	14.9	44	13.2	31	34	90	211	R
26	92	10	0.69	132	4.2	99	28	5	9.4	4.4	4.19	13.5	40	13	32	34	95	267	R
27										5.4	4.5	9.6	31	20.8	21	31	68	348	R
28	104	26	0.95	139	4.7	104	29	6	9.7	5	5.09	14	43	17.5	28	33	84	152	A
29										3.6	3.73	7.9	26	24.5	21	30	71	228	A

 © 2025. The Author(s).

Supplementary Table S2 *(Continued)*

Subject	Glu	Urea nit	Creat	Na	K	Cl	CO2	An gap	Ca	WBC	RBC	Hemoglobin	Hematocrit	RDW	MCH	MCHC	MCV	Plat	DOAC
30	77	15	0.84	138	4.8	102	25	11	9.3	7	5	14.4	44	14.8	29	33	88	299	A
31	76	13	0.59	138	4	103	25	10	8.8	4.7	4.42	13	39	13	29	33	88	255	A
32	111	20	0.64	142	4.5	102	30	10	10.4	10.8	4.33	13.4	40	13.7	31	34	92	207	R
33																			R
34	103	22	0.96	128	5.6	94	29	5	9.3	12.4	4.55	13.1	38	13.5	29	34	84	248	A
35	88	13	0.98	136	4.7	100	31	5	10.4	6.1	4.63	14.9	44	12.5	32	34	94	206	R
36	273	12	0.68	138	4.4	102	26	10	10.1	7.7	4.89	14.8	42	12.4	30	35	86	162	A

Supplementary Methods

Device Design

To maintain equivalent volumetric flow rates in each of the channels for fluid withdrawal through a single outlet, the fluidic resistance (R) was held constant (Eq. 1) for each of the 8 channels.

Eq. 1 $$R = \frac{12\mu L}{wh^3\left[1-\frac{192}{\pi^5 w}\sum_{n=1,3,5\ldots}^{\infty}\left(\frac{\tanh\left(\frac{n\pi w}{2h}\right)}{n^5}\right)\right]}$$

To avoid causing differences in platelet margination, effective hematocrit, or shear rate in each of the channels, the height and width of each channel were fixed, and thus the path length was required to remain constant from each inlet well to the focal imaging region. Serpentine paths were added for the wells closest to the imaging region to maintain equivalent resistance. Two-dimensional steady-state flow simulations (COMSOL 5.3a) were performed to verify the uniformity of flow in each of the channels.

[DOAC] Calculation

DOAC was calculated by scaling each patient's fibrin signal for their unmodified blood to historical data according to Eq. 2. FI_m is the effective measured fluorescence for the patient scaled to the reference dataset, FI_P is the mean raw fluorescence intensity of the patient's unmodified blood channels, FI_I is the mean fluorescence intensity of the fully inhibited channels, FI_R is the mean fluorescence intensity of the fully reversed channels, $FI_{DR,max}$ is the maximum mean fluorescence intensity in the reference IC50 dataset, and $FI_{DR,min}$ is the respective minimum.

Eq. 2 $$FI_M = \frac{FI_P - FI_I}{FI_R - FI_I} * \left(FI_{DR,max} - FI_{DR,min}\right) + FI_{DR,min}$$

The resulting FI_M from the equation can be used as an ordinate input to the equation of the three-parameter Sigmoid fit (hill slope =1) as the ordinate value of the IC50 curve from the in vitro dose-response model (►**Supplementary Fig. S3**), of the form.

Eq. 3 $$FI_M = FI_{DR,min} + \frac{FI_{DR,max} - FI_{DR,min}}{1+\frac{X}{IC50}}$$

Yielding the simple relation for the corresponding concentration that would produce that fluorescence, is described as follows in Eq. 4. This process is outlined graphically in ►**Fig. 2**, and in further detail in ►**Supplementary Fig. S1**.

Eq. 4 $$X = \frac{IC50\left(FI_{DR,max} - FI_M\right)}{\left(FI_M - FI_{DR,min}\right)}$$

LC-MS/MS Procedures

For the batch 1 samples, apixaban was extracted from the plasma samples as previously described (Pursley J, Shen JX, Schuster A, Dang OT, Lehman J, Buonarati MH, et al. LC-MS/MS determination of apixaban (BMS-562247) and its major metabolite in human plasma: An application of polarity switching and monolithic HPLC column. Bioanalysis. Future Science Ltd; 2014;6:2071–2082). Briefly, internal standard ($^{13}CD_3$

 © 2025. The Author(s).

Apixaban, Cayman) was added to 100 µL of plasma, 400 µL of 5 mM ammonium acetate was added, vortexed for 30 s, and centrifuged for 2 minutes at 10,000 g. The mixture was subjected to solid phase extraction using a Bond Elut, 100 mg, C18 cartridge (Agilent Technologies). The cartridge was conditioned with 1 mL methanol followed by 1 ml 5 mM ammonium acetate. Samples were loaded to the cartridge followed by washing with 1 mL solvent constituting methanol: 5 mM ammonium acetate (10:90 v/v). The cartridges were dried by vacuum for 15 minutes and samples eluted with 1 ml methanol. The extracts were dried under a stream of nitrogen and reconstituted in 250 µL solvent constituting 30:70 (v/v) methanol and 5 mM ammonium acetate). The samples were transferred to autosampler tubes and 20 µL of the samples were injected for analysis by ultra-performance liquid chromatography-tandem mass spectrometry (UPLC-MS/MS).

A Waters Acquity UPLC BEH column (2.1 × 150 mm) was used. Mobile phase A consisted of H_2O/B(95/5) + 0.5% HAc (pH adjusted to 5.7 w/ammonium hydroxide). Mobile phase B consisted of Acetonitrile/Methanol (95/5). The following gradient was run: 0 minutes 2% B; 5 minutes 2% B; 6 minutes 10% B; 12 minutes 60% B; 15 minutes 2% B; at a flow rate of 0.3 mL/min.· A Waters Xevo TQ-S instrument was run in positive ionization mode for the detection of the compounds. The mass transitions, cone, and collision energy used were: $^{13}CD_3$ Apixaban (464.27 > 202.97, 28, 32) Apixaban (460.2 > 443.1, 14, 22). A calibration curve was prepared in plasma from 0.012 to 3.12 µg/mL for Apixaban (Cayman).

For batch 2 samples, LCMS analysis was performed by Absorption Systems (Exton, PA). An aliquot of each human plasma sample (50 µL) was combined with 100 µL of acetonitrile in a 96-well plate. Calibration standards were prepared in mixed-gender human plasma obtained from BioIVT and immediately combined with 2 volumes of acetonitrile. Calibration standards ranged from 1 µg/mL to 0.3 ng/mL. Quality control samples were prepared ($n = 6$) at three calibration levels (0.3, 0.03, 0.003 µg/mL). After thorough mixing, the plate was centrifuged for 10 minutes at 3,000 rpm. An aliquot of supernatant was combined with 1 volume of water containing internal standard (0.1 µg/mL stable-isotopically labeled apixaban or rivaroxaban) and analyzed by LC-MS/MS.

For these samples, a Waters Acquity UPLC BEH Phenyl 30 column was used, with 25 mM ammonium formate buffer used as a mobile phase, pH 3.5. Aqueous reservoir (A) consisted of 90% water and 10% mobile phase buffer, and organic reservoir (B) consisted of 90% acetonitrile and 10% buffer. The following gradient was run: 0 minutes 1% B, 0.65 minutes 99% B, 0.75 minutes 99% B, 0.8 minutes 1% B, 1 minute 99% B, for a total run time of 1 minute at a flow rate of 0.7 mL/min.

A PE SCIEX API 4,000 mass spectrometer in multiple reaction monitoring modes, with a Turbo ion spray interface, was run for a total of 1 minute. The settings used are described in (►**Supplementary Table S1**).

LC-MS/MS Method Qualification

For the second batch of plasma samples, the LC-MS/MS method was qualified as follows: one eight-point standard curve and three levels of quality control (QC) samples with six replicates each were analyzed in the same run as samples were analyzed. At least 75% of standards were required to have accuracy within ± 15%, except at the LLOQ where ± 20% is acceptable. The intra-assay coefficient of variation of the replicate QC determinations was required to not exceed 15% and the accuracy of the mean value for each QC level was required to be within ± 15% of the theoretical value. At least 75% of quality control samples were required to have accuracy within ± 15%, and at least four of six QC determinations at each level were required to be available to calculate the statistics (►**Supplementary Fig. S8**).

ROC Analysis

Receiver operator characteristic (ROC) curves were calculated using GraphPad Prism for a >95 confidence interval with the Wilson/Brown method. For the purposes of the ROC analysis, "true positive" was defined as patient samples where the LC-MS/MS concentration was found to be higher than a specific threshold. Recommendations have been made by the International Society on Thrombosis and Haemostasis (ISTH) to consider the use of a reversal agent for drug concentrations of ≥30 ng/mL (~70 nM) for perioperative situations and ≥50 ng/mL (~120 nM) for major bleeding (27). To approximate these thresholds on a molar basis, 100 nM (Apix: 45.9 ng/mL, Riv: 43.6 ng/mL) and 60 nM (Apix: 27.6 ng/mL, Riv: 26.2 ng/mL) were used as threshold values.

 © 2025. The Author(s).
